# Polyphenol Rich Extract of *Garcinia pedunculata* Fruit Attenuates the Hyperlipidemia Induced by High Fat Diet

**DOI:** 10.3389/fphar.2016.00294

**Published:** 2016-08-31

**Authors:** Rahul Sarma, Sima Kumari, Ramakrishnan Elancheran, Meetali Deori, Rajlakshmi Devi

**Affiliations:** ^1^Biochemistry Laboratory, Life Sciences Division, Institute of Advanced Study in Science and TechnologyGuwahati, India; ^2^Drug Discovery Laboratory, Life Sciences Division, Institute of Advanced Study in Science and TechnologyGuwahati, India; ^3^Department of Zoology, Nalbari CollegeNalbari, India

**Keywords:** *Garcinia pedunculata*, polyphenol, antioxidant, hyperlipidemia, high-fat diet

## Abstract

Fatty foods, the most common diet today are the crux of many metabolic disorders which need urgent attention. *Garcinia pedunculata* Roxb. (GP, Clusiaceae) is a plant found available in Northeast (NE) region of India, is considered to have versatile therapeutic properties. The people of this region has been using dried pulp of GP fruit for the treatment of different stomach related diseases traditionally. This study aimed at evaluating the potential therapeutic action of the polyphenol-rich methanolic extract of the fruit in experimental induced obese rats. *In vitro* antioxidant and antidiabetic activity of GP extracts, i.e., fruit extract (GF) and seed extract (GS) were determined by using various methods viz., 1,1-diphenyl-2 picrylhydrazyl (DPPH), 2,2′-Azinobis (3-ethyl benzthiazoline-6-sulphonic acid) (ABTS^•+^), nitroblue tetrazolium (NBT) and α-glucosidase inhibition assay for detection of antihyperglycemic activity. *In vivo* antilipidemic and antiobesity activities were evaluated by administrating oral dose of GF for 60 days on a high-fat diet (HFD) induced hyperlipidemia in the rat. GF showed higher antioxidant activity than GS by DPPH radical scavenging (IC_50_ = 4.01 μg/ml), ABTS^•+^ (IC_50_ = 0.82 μg/ml), NBT (IC_50_ = 0.07 μg/ml) and also showed notable α-glucosidase inhibitory activity (IC_50_ = 19.26 μg/ml). Furthermore, GF treated rat revealed a reduction in the body weight (~60%), serum total cholesterol (33%), triglycerides (32%), low-density lipoprotein (38%) and liver biomarker enzymes after 60 days HFD fed animals. Simultaneously, GF supplementation significantly protected the HFD induced changes in hematological parameters. Histological observations clearly differentiate the structural changes in liver of HFD and GF treated group. This novel dietary lipid adsorbing agent of GF exhibited prevention of hyperlipidemia induced by HFD in the rat.

## Introduction

Plants represent one of the richest sources of natural diets which play pivotal roles in the treatment and prevention of various diseases. Recent studies indicate that the western style dietary patterns have contributed significantly to the development of cardiovascular diseases, dyslipidemia, cancer and diabetes (Fogli-Cawley et al., 2007; Kesse-Guyot et al., 2011; Maruthanila et al., 2014). Epidemiologic evidence also reveals that a high-fat diet is one of the contributing factors for the development of metabolic syndrome (MetS) both in animals and man (Despres and Lemieux, 2006; Bruce and Hanson, 2010; Yang et al., 2012). MetS includes different kinds of endocrine disturbances such as obesity, hyperlipidemia, dysglycemia, dyslipidemia, and hypertension, predisposing individuals to increase the risk for atherosclerosis, cardiovascular events and eventually type 2 diabetes mellitus (T2DM) (Moreira et al., 2014). Thus, several researchers have emphasized in dietary bioactive compounds that protect or mitigate the sufferings of different chronic diseases without side effects. Plant based dietary nutrients such as polyphenolic compounds (e.g., flavonoids, anthocyanins, and phenolic acids) were demonstrated to have potential health benefits for the treatment of hyperlipidemia. Flavonoids constitute a large proportion of those bioactive compounds (Martin and Appel, 2010). Fruits are one of the most important components of plant-based dietary nutrients that contain various bioactive nutraceuticals capable to enhancing body strength to fight various illnesses. The consumption of large quantity of fruits and vegetables are also beneficial for the treatment of chronic diseases (Liu et al., 2004; Estaquio et al., 2008). Since, ancient times various ethnically and topographically specific fruits are used as traditional medicine for treatment of different diseases including diabetes and obesity (Babio et al., 2009). In India, Ayurvedic medicines employ a wide range of locally harvested fruits to treat different health problems (Krishnaveni and Mirunalini, 2010).

*Garcinia pedunculata* Roxb. (GP), a semi-wild species of Clusiaceae family is an evergreen tree, endemic to the south eastern regions of Asia such as parts of Myanmar and north eastern parts of India. Traditionally, the GP fruit has been using by the people of Assam as medicine to treat different types of stomach related diseases (Sarma and Devi, 2015). This plant is exotic to the rest of the world and the global community at large fail to derive benefit from their potential bio defensive effects. Earlier reports have shown that Garcinia species played significant role for the treatment of different diseases such as diabetic, Alzheimer’s, and normal aging (Yamaguchi et al., 2000). Additionally, GP fruit is used as a garnish for curry and in some of the folklore medicine in India and contains 2-3% garcinol (Krishnamurthy et al., 1981). Recent years, the global scientific community has been interested to study and evaluate the medicinal potential of this plant and fruits. Garcinia is a rich source of secondary metabolites including xanthones, flavonoids, benzophenones, lactones, and phenolic acids with wide range of biological and pharmacological activities. (Jayaprakasha et al., 2006). Considering the importance of GP fruit as traditional herbal medicine, the objective of the study is to evaluate the potencial value of GP fruit to prevent hyperlipidemia.

## Materials and Methods

### Drugs and Chemicals

1,1-diphenyl-2 picrylhydrazyl (DPPH), 2,2′-Azinobis (3-ethyl benzthiazoline-6-sulphonic acid) liquid substrate (ABTS), trolox, ascorbic acid (AA), hydrogen peroxide (H_2_O_2_), Folin-Ciocalteu phenol reagent, nitroblue tetrazolium (NBT), superoxide dismutase (SOD), catalase (CAT), glutathione (GSH), catechin, quercetin, orlistat were obtained from Sigma-Aldrich Chemicals (St Louis, MO, USA). Other chemicals of analytical grade were purchased from Merck Limited (Mumbai, India).

### Plant Collection and Identification

A sample of fresh GP fruit was collected from Lakhimpur district of Assam, India(situated in between 27°14′–28°16′ North latitude and 94°07′–96°01′ East latitude), in the month of March 2015. The fruits were then sliced into small pieces and seeds were separated. The sliced small pieces of pulps and their seeds were dried up separately. The samples were authenticated by an expert Taxonomist, Department of Botany, Gauhati University. Herbarium was prepared and voucher specimen numbers (Acc. No. IASST/LSD/PM- 17A and IASST/LSD/PM- 17B) were deposited in medicinal and aromatic plant section, Life Sciences Department, Institute of Advanced Study in Science and Technology (IASST), Assam, India.

### Preparation of Extracts

The dried samples treated with sufficient amount of methanol (1000 ml) by maceration with continuous stirring for 3 days at room temperature (25 ± 2°C). The extracts were filtered through a cotton plug followed by Whatman No. 1 filter paper. Final extracts were concentrated through vacuum evaporation (Buchi R-210, USA) and all dried extracts of fruit (GF) and seed (GS) were stored at -20°C until used for further analysis (Sowemimo et al., 2015). The percentage yield was calculated 12-15% for GF and 6-8% for GS.

### Measurement of *In vitro* Antioxidant Activity

#### DPPH Radical Scavenging Assay

The DPPH (1,1-diphenyl-2 picrylhydrazyl) assay was done according to the method of Kumari et al. (2016) using UV spectrophotometer. The solution of DPPH in methanol (6 × 10^-5^ M) was prepared just before UV measurements samples were added to DPPH solution in 1:1 ratio followed by vortexing. The absorbance at 515 nm was measured at different time intervals. AA served as standard. The decreased absorbance of the DPPH solution at λ = 515 nm indicates an increase of the DPPH radical scavenging activity. Determination of DPPH scavenging concentration 50% (IC_50_) of an extract was performed with several serial dilutions of extracts. Percentage scavenging of DPPH by extract was calculated by applying formula:

(1) $$\left[\frac{\left({Absorbance}_{control}-{Absorbance}_{sample}\right)}{{Absorbance}_{control}}\right]\times 100.$$

#### ABTS^•+^ Scavenging Assay

The scavenging of ABTS^•+^ has been performed as described from the method of Walker and Everette (2009) with slight modification. Briefly, 100 ml stock solution of ABTS^•+^ (0.5 mM) was prepared by addition of 1 ml potassium persulfate (6.89 mM PBS, pH 8.0). The mixture was stored in the dark for 16 h. Ten micriliter of various dilutions of extracts were mixed with 190 μl of ABTS^•+^ in a 96-well microplate. The absorbance of decolorized ABTS^•+^ was measured at 734 nm and percentage of scavenging was calculated using the above Eq. (1). Trolox was used as reference compound.

#### Nitric Oxide (NO) Radical Scavenging Activity

Nitric oxide radical scavenging activity was measured according to the method of Kumari et al. (2016). NO radical were generated from sodium nitroprusside (SNP) solution. One milliliter of SNP (10 mM) was mixed with 1 ml of extracts in phosphate buffer (0.2 M, pH 7.4). The mixture was incubated at 25°C for 150 min. After incubation, 1 ml Griess reagent (1% napthalenediamine dichloride and 2% phosphoric acid) was added. The absorbance was measured at 546 nm and percentage of scavenging was calculated in the formula as shown above Eq. (1). AA used as a standard.

#### Hydrogen Peroxide (H_2_O_2_) Radical Scavenging Activity

The radical scavenging activity of the extracts against H_2_O_2_ was determined using the method of Ruch et al. (1989). H_2_O_2_ (43 mM) was prepared in 0.1 M phosphate buffer solution (pH 7.4). Samples (1 ml) were mixed with H_2_O_2_ solution (43 mM). After 10 min, the reaction mixture absorbance was measured at 230 nm. The phosphate buffer without H_2_O_2_ was used as blank. Trolox was used as reference compound. The percentage of scavenging was calculated using the above Eq. (1).

#### Reducing Power Assay

The reducing powers of the extracts were measured by the method of Kumari et al. (2016). Briefly, various concentrations of 0.2 ml of sample were mixed with 2.5 ml phosphate buffer (0.2 M, pH 6.6) and 2.5 ml of 1% potassium ferricyanide. After incubation at 50°C for 20 min, 2.5 ml of 10% trichloroacetic acid (10%) was added and the reaction mixtures were centrifuged at 4000 rpm for 10 min. Then 2.5 ml supernatant was collected and mixed with 2.5 ml distilled water of 0.5 ml ferric chloride (0.1%). The absorbance was measured at 700 nm. The increased absorbance of the reaction mixture indicated increasing reducing power. Trolox was used as a standard.

#### NBT Reducing Assay

Nitro blue tetrazolium reducing activity was measured by the method of Tiwari et al. (2011). In a 96-well plate containing 100 μl phosphate buffer (50 mM, pH 10) and an equal quantity of NBT (1 mM, prepared in the same buffer), 50 μl of extract was mixed and incubated for 15 min. A blank with extract in the absence of NBT was run to correct background absorbance. The reduction of NBT was measured at 560 nm using microplate multimode reader (Thermo Scientific, Varioskan flask) and the percentage of NBT reduction was calculated by applying formula:

$$\left[\frac{\left({Absorbance}_{control}-{Absorbance}_{sample}\right)}{{Absorbance}_{control}}\right]\times 100.$$

AA used as a standard.

#### Lipid Peroxidation (LPO)

Lipid peroxidation (LPO) induced by Fe^2+^ascorbate system in rat liver homogenate was estimated as thiobarbituric acid reactive substances (TBARS) by the method of Kumari et al. (2016). The reaction mixture contained rat liver homogenate 0.25 ml (10% w/v in 0.05 M phosphate buffer, pH 7.4), 0.1 ml tris-HCl buffer (150 mM, pH 7.2), 0.05 ml ascorbic acid (0.1 mM), 0.05 ml FeSO_4_.7H_2_O (4 mM), and 0.05 ml of fruit extract. The mixture was incubated at 37°C for 1 h and then 1.5 ml 2-thiobarbituric acid (TBA, 0.8% w/v), 1.5 ml acetic acid (20%) and 0.2 ml sodiumdodecyl sulfate (SDS, 8.1% w/v) were added to the reaction mixture. The mixture was made up to 4.0 ml with distilled water and heated for 60 min. After cooling with tap water, 1.0 ml distilled water and 5.0 ml of a mixture of *n*-butanol and pyridine (15:1, v/v) were added. The mixture was shaken vigorously and centrifuged at 5000 rpm for 10 min. After centrifugation, the optical density of the butanol layer was measured at 532 nm. Trolox was used as a standard.

### α-Glucosidase Inhibition Assay

α-Glucosidase inhibitory activities were evaluated according to the method described by Tiwari et al. (2011). Twenty microlitres of extract (10 mg/ml DMSO) was incubated with 50 μl of crude intestinal α-glucosidase for 5 min and then with 50 μl of substrate 5 mM *p*-Ni- trophenyl-α-D-glucopyranoside. The absorbance of all extrtacts were well measured with a microplate reader at 405 nm, while the reaction system without plant extract was used as control. The system without α-Glucosidase was used as blank, and acarbose was used as positive control. The enzyme inhibitory rates of samples were calculated as follows

$$Inhibition\%=\left[\frac{\left({Absorbance}_{control}-{Absorbance}_{sample}\right)}{{Absorbance}_{control}}\right]\times 100.$$

### Phytochemical Analysis

#### Total Phenolic Content

Total phenolic contents of the two extracts were determined by Folin-Ciocalteu method of Deori et al. (2014). Briefly, two extracts (0.5 ml) were mixed with Folin-Ciocalteu reagent (2.5 ml, diluted 10 times) and incubated for 2 min at room temperature followed by addition of sodium carbonate solution (2 ml, 7.5% w/v). The mixture was then allowed to stand for 30 min at room temperature and absorbance was measured at 765 nm. The amount of total phenolic content was calculated as a catechin equivalent from the calibration curve of catechin standard solutions and expressed as mg catechin/gm of extract.

#### Total Flavonoid Content

Total flavonoid content was estimated according to the method of Deori et al. (2014). Two milliliter of extract was mixed with 2 ml of AlCl_3_ in methanol (2%) in the GF and GS extracts. The absorbance was read at 415 nm after 10 min. Quercetin was used as a reference compound and the results were expressed as mg quercetin/g dry weight of the extract.

#### Total Anthocyanins Content

Total anthocyanins content in GF and GS extracts were determined as described by Giusti et al. (1999). Twenty-five micrometer potassium chloride solution (pH 1.0) and 0.4 M sodium acetate buffer (pH 4.5). The extracts were mixed directly with equal volumes of the two buffers separately. After ensuring through mixing, their absorbance was read at 510 and 700 nm, respectively (Multimode reader, Thermo Scientific, Varioskan flask). Data was expressed using molar extinction coefficient, the molecular weight of anthocyanins and an absorbance of A = [(A_510_-A_700_) pH 1.0-(A_510_-A_700_) pH 4.5] as milligrams of anthocyanins per 100 g extract.

#### Total Antioxidant Activity (TAA)

The antioxidant activities were measured with a photochem system (Analytik Jena AG, The Woodlands, TX, USA). The system based on photo chemiluminescence (PCL) for the quantification of antioxidant capacity of FPandSE. Free radicals (superoxide anion radicals) were generated by photochemical excitation followed by luminescence detection method (Popov and Lewin, 1999). The free radicals generated by the optical excitation of the photosensitizer substance were partly eliminated by the reaction of antioxidants in the sample to be analyzed. In a measurement cell, the luminescence of the detection material (luminol) generated by the remaining radicals was measured and thus the quantity of antioxidants present in the sample was determined by equivalents to ascorbic acid (Kalita et al., 2016). PCL can measure antioxidant activity in the nanomolar range. The reagent kits used for analysis were obtained from Analytik jena AG. Ten microliter of GF and GS extracts were used for each measurement.

### Mineral Content Analysis

Mineral content was analyzed with Atomic Absorption Spectrophotometer, SHIMADZU AAC-7000 analyzer (Deori et al., 2014). Extracts of GF and GS (100 mg) were digested with 3 ml concentrated nitric acid (65%) and 0.25 ml hydrogen peroxide until a transparent solution was obtained. Finally after digestion the volume was made upto 30 ml with distilled water. The instrument was calibrated with known standards and samples analyzed at corresponding wavelengths. Na and K were determined by Flame photometer, ELICO CL 378.

### *In vivo* Assay

#### Induction of Hyperlipidemia

The experiment was conducted using healthy male Wister albino rat (150-200 gm) in accordance with the internationally accepted guideline for experimental animal use and care, and the study was approved by the Institutional Animal Ethics Committee (IAEC; 1706/GO/C/13/CPCSEA). Animals were housed in individual polypropylene cages in an ambient temperature of 24 ± 3°C and relative humidity 45 ± 5% with a 12 h light-12 h dark cycle. They were fed pellet diet consisting of nitrogen free extract 51.65%, crude protein 21.36%, crude fat 10.63%, total ash 7.41%, moisture 6.32%, crude fiber 2.36%, calcium 1.75%, phosphorous 1.1%, water activity 0.23% per 100 g of the diet (collected from Nutrilab, Kolkata, India). After 1 week of acclimatization with free access to pellet diet and water, animals were used in the study. Rats fed with prepared high-fat diet (HFD) and water *ad libitum* for the period of 8 weeks. Composition of the HFD (g/kg diet) was according to the formula of Srinivasan et al. (2005) with some minor modifications and consisting of powdered normal pellet diet 375 g, lard 290 g, casein 265 g, corn oil 10 g, cholesterol 10 g, vitamin and mineral mixture 60 g, DI Methionine 03 g, yeast powder 01 g, and sodium chloride 01 g.

#### Acute Toxicity Studies

An acute oral toxicity study was performed as per Organisation for Economic Co-operation and Development (OECD 423) guidelines (acute toxic class method), albino mice (*n* = 6) of either sex selected by random sampling. The animals were kept fasting overnight and provided water only, and then the GF was administrated orally at 2000 mg/kg and observed for 15 days. In doing so, if the mortality rate was 2 out of 3, then the dose was considered as toxic. In case, the mortality was 1 out of 3, then the experiment needed to be repeated. If the mortality still continued, then low dose to be administered (Ecobichon, 1997).

#### Animal Treatment

In this study, a total of 24 rats were used and divided into four groups of 06 rats each as follows:

Group I: (Normal healthy control) fed with normal pellet diet and water *ad libitum* for 60 days.
Group II: (HFD control) fed with HFD for 60 days.
Group III: (Orlistat treatment group) fed with HFD for 60 days + from 15th day, Orlistat (30 mg/kg/p.o.) to 60 days.
Group IV: (Methanolic GF extract treated group) fed with HFD for 60 days + from 15th day, methanolic GF extract (200 mg/kg/p.o.) to 60 days.

The drug treatment continued from the whole experimental period with the extract being dissolved in 0.3% carboxymethyl cellulose (CMC) and then orally administered to the rats at an interval of 24 h. During the experimental period, the body weight of each rat was measured weekly upto 8 weeks. After completion of 8 weeks, the rats were kept under fasting for 12 h and then sacrificed; blood and tissues such as heart, liver, kidney, different fats, and muscles were collected for morphometric assessment and further analysis. The liver was washed with ice cold saline and preserved for histopathological analysis.

#### Effect of GF on Body Weight and Liquid Intake

Body weight and water intake measurements started from the first week of the study and continued for the entire experiment on the weekly basis of each rat.

#### Serum Biochemical Estimation

Blood was collected from the juglar vein (Tiwari et al., 2014) and allowed to clot. Serum was separated by centrifugation at 3000 rpm for 10 min for estimation of total cholesterol (TC), triglycerides (TG), and high-density lipoprotein-cholesterol (HDL-C) were estimated enzymatically using standard kits (Accurex Biomedical Pvt. Ltd., Thane, India), whereas low-density lipoprotein-cholesterol (LDL-C) were calculated by using the equation (Friedewald et al., 1972): LDL-C mg/dl = (TC-HDL-C- TG/5). Serum activity of liver function enzymes: alkaline phosphatase (ALP), aspartate transaminase (AST), and alanine transaminase (ALT), and the concentration of total protein, albumin, globulin, bilirubin, creatinine, and uric acid were estimated by using commercial kits (Aspen Pvt. Ltd., Delhi, India).

#### Measurement of Endogenous Antioxidants and Oxidative Stress Markers

Endogenous antioxidant, such as GSH by Ellman (1959); SOD by Marklund and Marklund (1974) and catalase (CAT) activity were determined by Goth (1991). TBARS was measured as a marker of LPO, according to the method of Ohkawa et al. (1979). Nitrate/nitrite levels were measured by using the method of Green et al. (1982).

#### Effect of GF on Fasting blood glucose (FBG)

Fasting blood glucose (FBG) was measured (8 h fast, blood collected by tail cut method) according to manufacturers’ recommendation using glucometer (Acc-check Active, Roche Diagnostic, Germany).

#### Hematological Analysis

Blood samples with EDTA were analyzed using established procedures and automated Swelab alfa hematology analyzer. Parameters that were recorded included Hemoglobin (Hb), Red blood cells (RBC), White blood cells (WBC), Platelets, Packed cell Volume (PCV), Mean corpuscular hemoglobin (MCH), Neutrophils, Lymphocytes, Monocytes, and Eosinophils.

#### Histopathological Analysis

After the collection of blood, all the animals were euthanized for gross pathological examinations of the liver organ. Then it was planned to perform the histopathological examination for group I, II, III, and IV separately. The selected liver organs were fixed in 10% neutral buffered formalin. The histological slides were prepared by the standard protocol of dehydration and paraffin embedding (Carleton, 1930). Sections 5 μm were cut and stained with hematoxylin and eosin. Afterward, the sections were observed underphase contrast microscope (10× magnification, Zeiss phase contrast microscope).

### Statistical Analysis

All the results are expressed in mean ± SEM. *In vitro* antioxidant values of GF and GS were tested by using one-way analysis of variance. Data from each group I, II, III, and IV were tested by two-way analysis of variance followed by Dunnett’s multiple tests. All statistical analysis was performed by using Statistical Package for the Social Sciences (SPSS) version 16.0, Chicago, USA. *P*-value of <0.05 was considered as statistically significant.

## Results

### *In vitro* Antioxidant Assays

#### DPPH, ABTS^•+^, H_2_O_2_ Free Radical Scavenging Assay

Two extracts showed a concentration dependent scavenging activity against DPPH, ABTS**^•+^**, and H_2_O_2_ radicals. IC_50_ values of the GF and GS against DPPH free radical were found to be 4.01 and 25.81 μg/ml, respectively, which could be comparable with the AA with an IC_50_ value 0.33 μg/ml. In the case of ABTS^•+^, the IC_50_ values were 0.82 and 1.14 μg/ml, respectively, while that of trolox showed an IC_50_ value of 5.62 μg/ml. In H_2_O_2_, the IC_50_ values were 2.19 for GF and 2.8 μg/ml for GS, and standard trolox showed an IC_50_ value 8.18 μg/ml.

#### LPO and NO Inhibition Assay

In the case of LPO, IC_50_ values of GF were found to be 30.36 and GS 34.6 μg/ml, and that of AA is 18 μg/ml. The NO radical also scavenged by two extracts and their 50% inhibition was 1.48 and 4.76 μg/ml, respectively and the AA was 8.84 μg/ml.

#### Reducing Power Ability and NBT Reducing Assay

The reducing power ability of IC_50_ values of GF were 6.24 and GS were 8.5 μg/ml, and the trolox was 2.43 μg/ml. Regarding NBT reducing assay IC_50_ values of GF and GS were 0.07 and 1.18 μg/ml, respectively and the standard AA was 0.03 μg/ml.

#### α-Glucosidase Inhibition Assay

The α-Glucosidase inhibitory activities of GF and GS with IC_50_ values were19.26 and 24.87 μg/ml, respectively. The effectiveness of enzymatic inhibition of the extracts was determined by calculating IC_50._ The lower the value, the higher the quality of enzymatic inhibition.

#### Phytochemical Analysis

##### Total Phenolic, Flavonoid, Anthocyanins, and Total Antioxidant Content of GF and GS

Total phenolic content quantified in GF and GS extracts were 5.86 ± 0.04 and 4.45 ± 0.02 mg catechin/gram, respectively. Flavonoid content of GF was 5.60 ± 0.14 and GS 5.48 ± 0.04 mg quercetin/gm. The anthocyanins content in GF and GS were 6.67 ± 0.03 and 1.66 ± 0.02 mg/100 gm. The TAA in the GF and GS were quantified in equivalents to AA. GF and GS were 504 ± 3.2 and 362 ± 2.3 nmol/gm, respectively.

#### Mineral Content of GF and GS

Twelve minerals including two major elements (K and Na) were tested in respect of GF and GS separately. The values of mineral content so determined of GF and GS were 67.37 ± 0.55 and 61.06 ± 0.20 in K, 1.0 ± 0.07 and 1.0 ± 0.03 in Na, 3.5 ± 0.2 and 2.93 ± 0.03 in Fe, 0.39 ± 0.01 and 0.30 ± 0.014 in Cu, 0.23 ± 0.04 and 0.13 ± 0.03 in Mn, 0.69 ± 0.30 and 0.73 ± 0.001 in Zn, 0.002 ± 0.0001 and 0.002 ± 0.0003 in Cd, 0.03 ± 0.002 and 0.04 ± 0.002 mg/100 g in Cr, respectively. But, in the case of Ni, Co, Pb, and Se mineral values could not be detected.

### Acute Toxicity Effect

The results of the acute oral administration of GF extract given at a dose of 2000 mg/kg to the mice indicated no mortality upto 15 days. Similarly by the administration of GF extract at a dose of 2000 mg/kgdidnot show any change in general behavior or lethality. So 1/10th of non-lethal dose (200 mg/kg) was selected for *in vivo* study.

### Effect of GF on Morphometric Parameters

**FIGURE 1:**
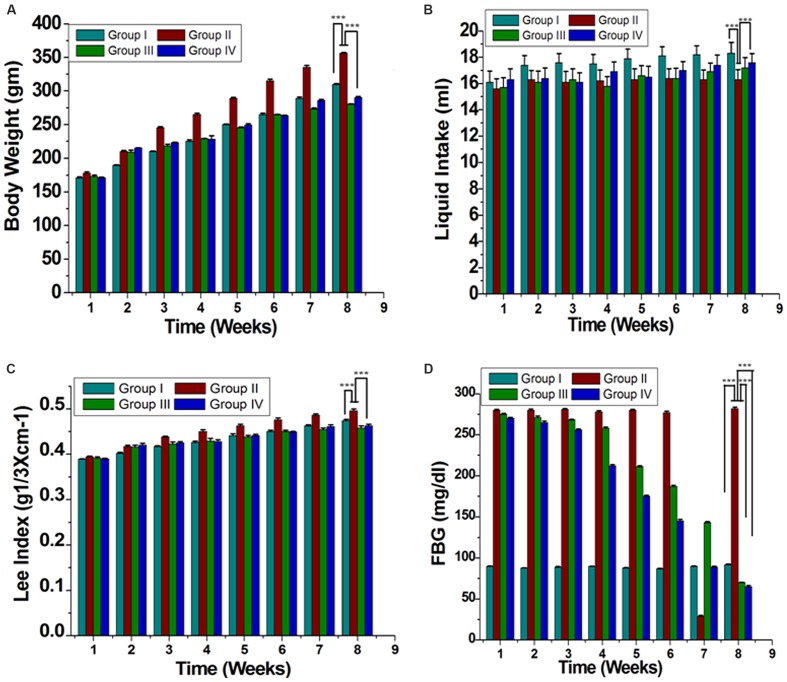
**Effect of ME of GF and GS on morphometric parameters (A) Body weight of evaluation, (B) Liquid intake, (C) Lee index [body weight (g) ^1/3^/naso-anal length (cm)], and (D) Fasting blood glucose (FBG) are shown.** All the results were expressed in mean ± SEM. ^∗∗∗^*P* < 0.05 in the comparison between different groups.

Initial average body weights of rats were 151.2 ± 0.44 g, and were not significantly different among four groups. After 8 weeks, group II increased body weight compared with that of group I (**Figure 1A**). Intake of GF extract reduced body weight for the group IV by ~60% than group II (**Figure 1A**). Lee-Index was more in group II than GF treated group (**Figure 1C**). Liquid consumption by rats in group I (18.3 ± 0.83 ml day^-1^ per rat) was the highest whereas group II (16.3 ± 0.76 ml day^-1^ per rat) was the lowest (**Figure 1B**). The average consumption of the group IV was 17.6 ± 0.69 ml day^-1^ per rat. Additionally, GF consistently decreased white adipose tissue depots in group IV with no effect on group I (**Table 1**). GF had no effect on brown adipose tissue, skeletal muscle or liver relative masses (**Table 1**).

**Table 1:** Morphological parameters and lipid profiles of 8 weeks treatment of control and HFD-obese rats.

	Group I	Group II	Group III	Group IV
**Morphological parameters (g/100 g BW)**				
Retroperitoneal fat	0.60 ± 0.11^a^	2.47 ± 0.12^c^	0.51 ± 0.06^a^	1.43 ± 0.11^b^
Periepididymal fat	0.53 ± 0.09^a^	3.28 ± 0.12^c^	0.52 ± 0.08^a^	1.79 ± 0.11^b^
Mesenteric fat	0.51 ± 0.15^a^	2.72 ± 0.05^c^	0.46 ± 0.07^a^	1.37 ± 0.08^b^
Interscapular brown fat	0.06 ± 0.07^a^	0.15 ± 0.04^a^	0.06 ± 0.04^a^	0.15 ± 0.05^a^
Liver	4.81 ± 0.08^c^	2.87 ± 0.05^a^	2.81 ± 0.15^a^	3.23 ± 0.1^b^
Heart	0.38 ± 0.11^a^	0.36 ± 0.05^a^	0.35 ± 0.04^a^	0.48 ± 0.05^a^
Kidney	1.23 ± 0.02^b^	0.96 ± 0.23^a^	0.94 ± 0.06^a^	1.26 ± 0.14^b^
Soleus muscle	0.03 ± 0.005^a^	0.04 ± 0.005^a^	0.03 ± 0.03^a^	0.03 ± 0.04^a^
Gastrocnemius muscle	0.46 ± 0.11^a^	0.43 ± 0.07^a^	0.45 ± 0.08^a^	0.42 ± 0.05^a^
**Liver lipid profile (mg/g)**				
Triglycerides	3.9 ± 1^a^	6.4 ± 0.41^b^	3.8 ± 0.92^a^	5.7 ± 0.23^b^
Total cholesterol	1.2 ± 0.56^a^	1.6 ± 0.32^a^	1.4 ± 0.47^a^	1.3 ± 0.72^a^
**Skeletal muscle lipid profile (mg/g)**				
Triglycerides	4.2 ± 0.45^b^	8.4 ± 0.4^c^	3.8 ± 0.7^ab^	2.8 ± 0.75^a^
Total cholesterol	1.1 ± 0.72^a^	1.3 ± 0.2^a^	1.2 ± 0.51^a^	1.3 ± 0.64^a^
**Oxidative stress markers**				
Serum SOD (% SOD)	34.3 ± 1.2^b^	25.8 ± 2.2^a^	41.6 ± 1.9^c^	39.4 ± 3.1^c^
Serum GSH (μg/ml)	1220.1 ± 14^b^	1130.5 ± 32^a^	2430.2 ± 19^d^	2192.6 ± 29^c^
Serum catalase (unit mg protein)	112.6 ± 16^c^	67.3 ± 4.2^a^	93 ± 6.8^b^	92.8 ± 5.06^b^
Serum TBARS (nmole/ml)	20.2 ± 1.9^b^	22.8 ± 2.3^c^	18.6 ± 2.7^a^	17.9 ± 4.1^a^
Serum NO (μM/ml)	22.9 ± 2.7^a^	35.3 ± 2.1^c^	24.8 ± 3.2^b^	24.2 ± 1.5^b^

*Data represent mean ± SEM (n = 6), comparison between group pairs were performed by ANOVA, using Duncana test. Different superscript letters (a,b,c,d) indicate statistical difference (P < 0.05) between the groups.*

### Effect of GF on Biochemical Parameters

**FIGURE 2:**
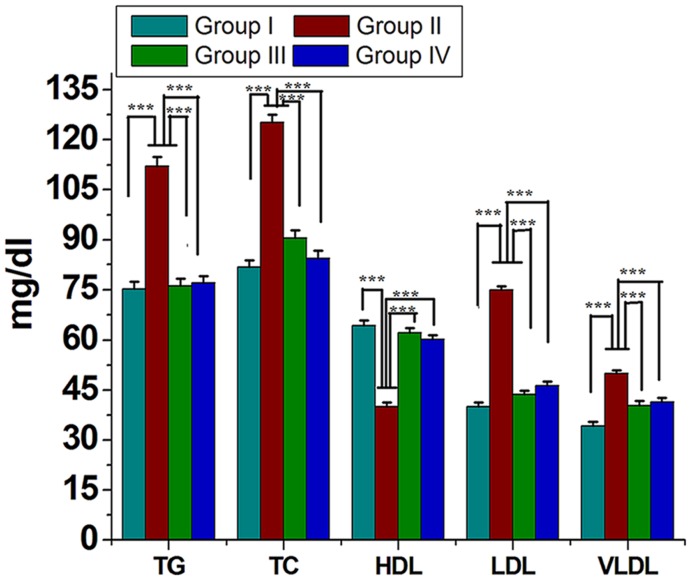
**Effect of ME of GF and GS on lipid profile of experimental rats.** Values are mean ± SEM. ^∗∗∗^*P* < 0.05 in comparison between different groups.

**FIGURE 3:**
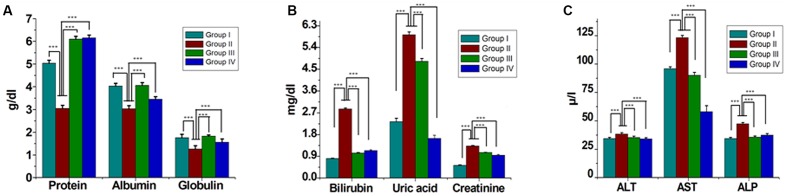
**Effect of ME of GF and GS on serum biochemical parameters.** Serum levels of **(A)** Proteins, albumin, globulin **(B)** bilirubin, uric acid, creatinine **(C)** alanine transaminase (ALT), aspartate transaminase (AST), and alkaline phosphatase (ALP). Values are mean ± SEM. Means. ^∗∗∗^*P* < 0.05 in the comparison between different groups.

Serum lipids (TC, TG, LDL-C, and VLDL-C) were increased significantly (*P* < 0.05) in group II animals in comparison to that to that of group I (**Figure 2**). However, these parameters were decreased significantly (*P* < 0.05) in group IV (about 32% for TG and 38% for LDL-C). HDL-C, a beneficial lipoprotein, was decreased in group II as compared to that of group I, and the results reversed on group IV (*P* < 0.05; **Figure 2**). Hepatic as well as skeletal muscle TG accumulation were ~ twofold higher in group II versus group I rats, but treatment with GF extracts normalized these levels. There were no significant differences in TC content in the liver or skeletal muscle between any treatment groups (**Table 1**). Treatment with GF extract did not have any significant adverse effect on hepatic biomarker enzymes ALT and ALP while it significantly decreased the activity of AST enzyme (**Figure 3C**). Moreover, GF extract did not alter the level of albumin, which is one of the major tests to assess the liver damage (**Figure 3A**). Kidney function marker creatinine and serum uric acid were shown in **Figure 3B**. In group II, HFD increases the level of uric acid and creatinine. The levels of these markers were significantly improved by supplementation with GF extract in group IV and were comparable to the normal control group I (**Figure 3B**).

### Effect of GF on Endogenous Antioxidants and Oxidative Stress Marker

Lipid peroxidation level was elevated significantly in group II compared to group I. Administration of GF (200 mg/kg) reduced the LPO level significantly on the 8th week (**Table 1**). The GSH level was found to be low in the serum of group II (*P* < 0.05) while in the group IV the levels increased on the 8th week when compared with the group I (*P* < 0.05; **Table 1**). SOD values in rats treated with GF extract was significantly higher (*P* < 0.05) on the 8th weeks treatment (**Table 1**). In serum of HFD rat, NO level was found to be more as compared to that of control (**Table 1**). GF and orlistat treated groups showed a lower level of NO as compared to the HFD induced group.

### Effect of GF on FBG

Blood glucose level significantly increased in the case of group II compared to group I. Treatment of group III with orlistat restored blood glucose to almost normal level. Similarly, a similar effect was observed in rats administration of GF of GP (group IV) at a dose of 200 mg/kg (**Figure 1D**).

### Effect of GF on Hematological Parameters

A significant difference was found with hematological parameters. Compared to that of the control, WBC, PLT, MCV, MCH, N, M, and N/L rats were significantly (*P* < 0.05) increased by HFD treatment but the values of WBC, PCV, MCV, MCH, and E count were significantly decreased by GF treatment (**Table 2**).

**Table 2:** Effect of GF on the hematological parameters of rats.

Hematological parameters	Group I	Group II	Group III	Group IV
RBC (millions/cmm)	6.84 ± 0.03^c^	2.24 ± 0.08^a^	6.2 ± 0.64^b^	7.36 ± 0.03^d^
WBC (/cmm)	8,800 ± 1^h^	22,700 ± 8.3^j^	8,900 ± 4.9^i^	4,500 ± 3.2^c^
PLT (/cmm)	414,000 ± 3^a^	888,000 ± 3^k^	513,000 ± 5^d^	6,88,000 ± 0.5^j^
Hb (gm%)	12.5 ± 0.36^a^	12.1 ± 0.25^a^	12 ± 0.45^a^	13.8 ± 0.49^b^
PCV (%)	38 ± 0.1^g^	19.9 ± 0.75^a^	42 ± 0.4^h^	35.5 ± 0.03^e^
MCV (cuμ)	55.5 ± 0.57^e^	88.5 ± 0.25^g^	57.1 ± 0.40^f^	48.3 ± 0.26^d^
MCH (pg)	18.3 ± 0.25^b^	54.0 ± 0.46^c^	18.8 ± 0.45^b^	16.4 ± 0.55^a^
N (%)	14 ± 0.60^a^	20 ± 0.64^c^	16 ± 0.35^b^	14 ± 0.75^a^
L (%)	77 ± 0.32^e^	70 ± 0.46^a^	77 ± 0.85^e^	80 ± 0.26^g^
M (%)	3 ± 0.26^b^	8 ± 0.2^d^	4 ± 0.58^c^	3 ± 0.26^b^
E (%)	6 ± 0.17^e^	6 ± 0.34^e^	4 ± 0.3^c^	4 ± 0.32^c^
N/L	0.18 ± 2^b^	0.28 ± 1.5^e^	0.20 ± 0.37^d^	0.17 ± 1^a^

*Here N, neutrophils; L, lymphocyte; M, monocyte; E, eosinophils; PLT, platelet and N/L, neutrophil/lymphocyte ratio.Values are presented as mean ± SEM (n = 6). Means within a row with unlike superscript letters a,b,c,d,e,f,g,h,…etc., differs significantly (P < 0.05). One way ANOVA followed by Dunnett’s multiple comparison was performed to analysis this data set.*

### Histopathological Changes

**FIGURE 4:**
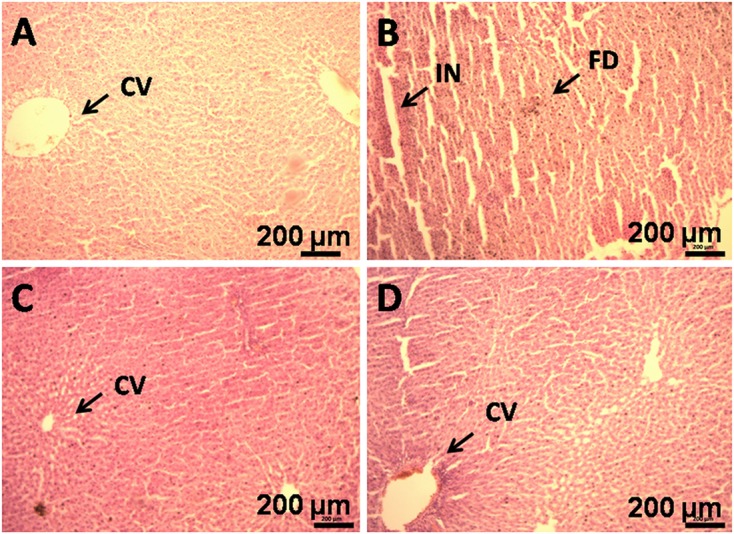
**Histopathology of liver of HFD rats supplemented with GF extract for 8 weeks. (B)** Haematoxylin and eosin staining of liver section showing hepatocytes with enlarge fat droplets (marked as “FD”), inflammatory cell infiltration (marked as “IN”) (X10). **(A)** Represents normal sized central vein (CV). **(C)** Represents orlistat (30 mg/kg) treated liver section, showing development in the hepatic structure and **(D)**. It is the GF (200 mg/kg) treated liver section, showing improvement in the hepatic cell structure.

Histological evaluation of liver tissue showed negligible fat droplets accumulation without inflammatory cells in group I (**Figure 4A**). HFD feeding resulted in the accumulation of fat droplets in the hepatocytes with sinusoids dilation and increased inflammatory cell infiltration in group II (**Figure 4B**). GF supplementation reduces the fat droplets and infiltration of inflammatory cells in group IV (**Figure 4D**).

## Discussion

*Garcinia pedunculata* Roxb. is used traditionally in many places for treatment of different kinds of diseases like diabetes, cardiovascular, inflammation, stomach related diseases etc., without any scientific basis. For this reason, we have screened out the GP extract to evaluate its antioxidant and antihyperlipidemic activities. During the study it revealed that GF extract of GP showed high antioxidant activity than that of GS. Herein, GF scavenges DPPH, ABTS**^•+^**, NBT, NO, H_2_O_2_, and caused inhibition of LPO in an efficient way. Different studies showed that free radicals and other reactive oxygen species are considered to be important causative factors in the development of diseases such as neurodegenerative diseases, cancer and cardiovascular diseases (Dhalla et al., 2000). A set of endogenous antioxidant enzymes such as GSH, SOD, and CAT play an important role in the elimination of ROS and protect cells against the deleterious effects of oxidative stress (Noeman et al., 2011). In this study, the HFD induced rat decreased the GSH, SOD, and CAT activities and increased the malondialdehyde (MDA) level. However, treatment of GF extract showed the increase in the GSH, SOD, and CAT levels with decreased MDA level. Additionally, GF contained various phytoconstituents, such as flavonoids, anthocyanins, phenolic compounds. Herein, GF was capable of inhibiting the α-glucosidase enzyme activity and thus showed significantly reduced postprandial plasma glucose levels and suppression of post prandial hyperglycemia. Many reports suggested that any change in the normal weight of human body leads to abnormal functions (Cui et al., 2011; Ansari et al., 2012). Nevertheless, it is assumed that the HFD induced rats are a useful model compatible to dietary fat in human (Cui et al., 2011; Noeman et al., 2011; Ansari et al., 2012). In the present study when the GF extract was administered in HFD induced rats, the body weight reduced significantly. Further, the Lee obesity index, a predictive marker of percentage body fat in rats, dramatically decreased in the GF treated group as shown in **Figure 1C**, which indicated that the fat content of HFD + GF fed rats had decreased. Moreover, quantitative data showed that the adipose tissues content decreased with GF treatment (**Table 1**).

Different studies revealed that the hematological system has the higher predictive value of any abnormal toxicity indications in human and the increase in the production of WBC and its differentials is considered as a marker of stress (Ashafa et al., 2009). In this study, the significant changes in the level of WBC and differentials including platelets and its indices neutrophils and monocytes suggested the toxicity of HFD. The test also showed noticeable haemolytic changes of the rats on RBC, MCV, and MCH in the HFD. Herein, when the GF extract was applied on the rats, the reduction of abnormal signs and symptoms in hematological parameters were clearly visible and significant. Thus, it indicated that GF extract had contribution to the reduction of any abnormal signs and symptoms in the hematological effects. Uric acid was the biomarker of kidney function and retention of these products in the body indicated renal damage (Odutola and Co Zaria, 1992; Newman and Price, 1999; Johnson et al., 2013). In the present study, the level of serum uric acid was elevated in the HFD induced rats. Whereas, after treatment with GF extract, the uric acid level significantly decreased (*P* < 0.05) in the HFD induced rats. It also showed that the levels of total proteins were decreased in HFD fed rats when compared to other rats with the normal diet. The decreased level of total protein in HFD fed rats might be due to the reduction in protein synthesis for high-calorie lipid diet. It was noteworthy that the administration of GF extract on the HFD induced rats could significantly restored the protein levels.

The liver biomarker enzyme such as AST, ALT, and ALP are the indicators of liver function (Han et al., 2012). ALP is indication enzyme found in the cell membrane of the liver and the elevation of this enzyme indicates primary hepatic disease (Han et al., 2012). Whereas ALT and AST are leakage enzyme, and their elevation indicates significant hepatocellular damage (Chapman and Hostutler, 2013). In the present study, the levels of these enzymes were found significantly increased in the HFD rats. Whereas, after the administration of GF extract in the HFD rats, the biomarker enzymes showed to have decreased which may account for the protective effect on liver disorders. Moreover, histological examination also revealed that HFD feeding resulted in the accumulation of fat in the hepatocytes with sinusoids dilation and increased inflammatory cell infiltration. However, the GF extract treated rats noticeably attenuated the fat droplets and infiltration of inflammatory cells of the liver (**Figure 4**).

Several reports suggested that elevated levels of plasma TG, LDL, and VLDL cholesterol constituents increase the risk factor for cardiovascular diseases, hypertension, obesity, and diabetes mellitus (Zicha et al., 1999; Lichtenstein et al., 2006; Shen, 2007; McBride, 2008). In the present study, HFD induced rats caused a significant elevation in the level of lipid constituents in the serum and decreased HDL level. Whereas, treatment of GF extract showed significant (*P* < 0.05) decrease in the levels of LDL cholesterol and VLDL cholesterol along with significant increase in HDL cholesterol level. Herein, TG level was noticeably higher in HFD induced rats than the control group but after treatment with GF extract and also the TG level was brought back to normalcy. Taken all together, this study strongly suggested that the GF showed potent antioxidant activity as well as efficient way to cure hyperlipidemia.

## Conclusion

The result revealed that GP had beneficial antioxidant properties and the GP treatment attenuated the hyperlipidemia as well as oxidative stress. Thus it may be concluded that the GP treatment is the efficient way to cure the hepatic steatosis and hyperlipidemia. However, a further study can be conducted to examine the ability of GP for potent drug discovery.

## Author Contributions

RS conceived and designed the experiment. RS, SK, RE, and MD performed the experiment. RS analyzed the data. RS wrote the manuscript. RD have done a critical revision of the manuscript for important intellectual content. RD has been the corresponding author throughout the writing process. All authors have contributed to the final version and approved the final manuscript.

## Conflict of Interest Statement

The authors declare that the research was conducted in the absence of any commercial or financial relationships that could be construed as a potential conflict of interest.
